# Evidence in Orthodontics related to qualitative research

**DOI:** 10.1590/2177-6709.23.4.064-071.oar

**Published:** 2018

**Authors:** Anderson Barbosa de Almeida, Isabel Cristina Gonçalves Leite, Girlene Alves da Silva

**Affiliations:** 1 Universidade Federal de Juiz de Fora, Faculdade de Medicina, Departamento de Saúde Coletiva, Programa de Pós-graduação em Saúde, ênfase em Saúde Brasileira (Juiz de Fora/MG, Brazil). Universidade Federal de Juiz de Fora Universidade Federal de Juiz de Fora Faculdade de Medicina Departamento de Saúde Coletiva Juiz de ForaMG Brazil; 2 Universidade Federal de Juiz de Fora, Faculdade de Medicina, Departamento de Saúde Coletiva (Juiz de Fora/MG, Brazil). Universidade Federal de Juiz de Fora Universidade Federal de Juiz de Fora Faculdade de Medicina Departamento de Saúde Coletiva Juiz de ForaMG Brazil

**Keywords:** Orthodontics, Qualitative research, Research methodology

## Abstract

**Introduction:**

Research in Orthodontics has historically followed the positivist model based on the direct relationship of cause and effect between diseases and their specific etiological factors. Despite the objectivity and the great potential of statistical procedures, quantitative methods have progressively been sharing space with other models that can encompass the multiplicity of factors that affect the health-disease process, which until such time was reduced to its biological dimension.

**Objectives:**

This study aims, through an integrative review, to identify orthodontics articles published over a 10-year period that have used, exclusively or not, some method of qualitative research, and analyze the main aspects of their content.

**Methods:**

A survey was performed on Pubmed, Medline, Scopus, and Lilacs databases from 2007 to 2016 with a focus on the applicability of the qualitative methodology in orthodontic research.

**Results:**

The 27 articles selected showed a trend to increase in publications, with the most recent four years concentrating almost 60% of them. Most studies were from Europe, particularly the UK, and the more frequent study objectives were related to the perception of people about the reasons for orthodontic treatment, about the aesthetic and psychosocial impact of malocclusion or orthodontic treatment, and the implications of these factors for their quality of life.

**Conclusions:**

Because of its potential to explore behaviours and socio-cultural attitudes sustained in subjectivity, qualitative research offers new possibilities for orthodontic studies and can be used in an exclusive or complementary way in relation to quantitative methods.

## RESUMO

**Introdução:**

a pesquisa em Ortodontia tem, historicamente, seguido um modelo baseado na relação direta de causa e efeito entre doenças e seus fatores etiológicos específicos. Apesar da objetividade e do grande potencial de procedimentos estatísticos, métodos quantitativos têm, progressivamente, compartilhado espaço com outros modelos que podem englobar a multiplicidade de fatores que afetam o processo saúde-doença, o qual, até pouco tempo, era reduzido à sua dimensão biológica.

**Objetivos:**

esse estudo objetivou, por meio de uma revisão sistemática integrativa, identificar como tem sido aplicada a metodologia qualitativa em Ortodontia nos últimos 10 anos.

**Métodos:**

foi realizado um levantamento nas bases de dados Pubmed, Medline, Scopus e Lilacs, no período compreendido de 2007 a 2016, com um enfoque na aplicabilidade da metodologia qualitativa em pesquisas em Ortodontia.

**Resultados:**

os 27 artigos selecionados mostraram uma tendência ao aumento das publicações, com os últimos quatro anos concentrando quase 60% delas. A maioria dos estudos foi realizada na Europa, particularmente no Reino Unido, e os objetivos mais frequentes nos estudos foram relacionados à percepção das pessoas quanto aos motivos para o tratamento ortodôntico, o impacto estético e psicossocial da má oclusão e do tratamento ortodôntico, e sua relação com a qualidade de vida.

**Conclusões:**

devido ao seu potencial para explorar comportamentos e atitudes socioculturais sustentados na subjetividade, a pesquisa qualitativa oferece novas possibilidades para estudos ortodônticos e pode ser utilizada de forma exclusiva ou complementar aos métodos quantitativos.

## INTRODUCTION

Research in orthodontics has historically followed the positivist model based on the direct relationship of cause and effect between diseases and their specific etiological factors that can cause structural and physiological changes in the human body. Such thinking has fostered a conception of scientific rigor for quantitative research so great that it became hegemonic in the specialty.^1^^,^^2^

Despite the objectivity and the great potential of statistical procedures, quantitative methods have been progressively sharing space with other models, due mainly to the former’s inability to encompass the multiplicity of factors that affect the health-disease process, which until that time was reduced to its biological dimension.^3^^-^^5^

The recognition of the multifactorial nature of malocclusion^6^ highlights the importance of a greater effort to understand the variability with which similar stimuli can be interpreted by different individuals. In addition to its functional aspects, its influence on behavioral, emotional, and psychosocial aspects has been gaining increasing relevance in modern society, particularly due to its aesthetic implications.^7^^,^^8^

After an assessment of systematic reviews and randomized controlled trials, O’Brien^9^ states that the results of orthodontic research are confined to values and differences that are much more relevant for orthodontists than for their patients, which prompts the speculation that the planning of actions mediated by eminently quantitative data in the evaluations of the health-disease process may not meet the needs of the individuals involved. In pointing out the limits of the quantitative approach, the author indicates the need for closer ties with other research approaches that can comprehend the multifaceted phenomenon of the health-disease process.

In health field, a variety of objects are being explored from the perspective of qualitative research approaches because they contain elements that require an understanding of what constitutes the imagery of individuals and their relationships with the environment.^10^ Thus, Newton^1^ emphasizes that qualitative methods explore the complexity of behavioral contexts related to health and are useful for identifying subjective meanings of social phenomena and processes involved in health care, being essential in the interaction between patient and professional.

Unlike past eras when qualitative research articles were rejected for being considered non-scientific, in recent years, qualitative research studies have become well accepted by scientific journals.^10^^,^^11^

Thus, the present study aims to identify, through a systematic integrative review, how the qualitative methodology has been applied in orthodontic studies over a period of 10 years (2007-2016), analyzing the main aspects of their content such as frequency, origin, objects of study, and methods used.

## MATERIAL AND METHODS

**Figure 1:**
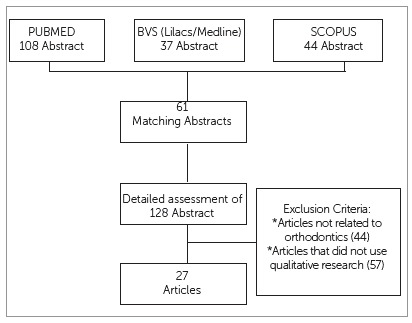
Search strategy for identification of studies.

A search for articles was conducted in the following databases: Pubmed, Medline, Scopus,and Lilacs, over the ten-year period from 2007 to 2016, with the initial question: “*How has qualitative methodology been applied in orthodontic research?*” The search terms utilized were: “*orthodontics*”,”*qualitative research*”, and “*qualitative methodology*”, which were cross-searched. The initial list of articles was submitted to analysis by two previously trained independent evaluators, who applied inclusion criteria to determine the final sample of articles, evaluated by their title and abstract. If there was any disagreement between the evaluators’ results, a third evaluator was consulted, reading the full version of the article (Fig 1).

The following inclusion criteria were used to select articles: studies written in English, Spanish, and Portuguese, published between January 2007 and December 2016; studies in orthodontics that might have used qualitative research methodology exclusively, or not. The ten-year period was used in order to estimate and assess the expansion of the importance of specific health areas through the profusion of scientific data.^12^

Given the diversity of age group categories from the studies reviewed, for the purposes of categorizing the participants as children, adolescents, and adults, the World Health Organization classification criterion was used, which establishes adolescence as a fundamentally biological process, during which cognitive development and the structuring of personality accelerates, and which would include the ages from 10 to 19 years, and thus identify children and adults as the individuals situated respectively below and above this range.^13^

## RESULTS

A summary of the results of the 27 selected articles is shown in Table 1, which presents characteristics related to the research objective and findings.

**Table 1:** Descriptive summary of the main characteristics of the reviewed articles.

Author (year) / nationality / periodical	Objectives		Methodological design / Number and age group of participants	Conclusions
Abed Al Jawad et al^28^ (2012) / United Kingdom / European Journal of Orthodontics	Elucidate the changes in the diets of patients during the early stages of orthodontic treatment with braces and identify factors influencing these changes in behavior.		Qualitative / Semi-structured interviews / 10 / Adolescents	Two main topics were presented: experience with pain and behavioral changes in response to the use of braces.
Bhamrah et al ^38^ (2015) / United Kingdom / American Journal of Orthodontics and Dentofacial Orthopedics	Investigate the information that orthognathic patients share and discuss with peers away from the clinical environment, to provide better information for orthognathic patients.		Qualitative / 1912 posts on internet forums / Adults	The study shows that orthognathic patients seek further information on the treatment, suggesting a possible gap in communication between patient and professional. Therefore, physicians should advise their patients so that they have access to relevant content.
Cirgic et al ^36^ (2015) / Sweden / Orthodontics and Craniofacial Research	Explore and describe experiences of adolescents in treatment with removable functional appliances.		Qualitative / Unstructured interviews / 21 / Adolescents (11-15 years old)	The experiences of adolescents in using removable functional appliances seem to be highly diversified, and the dentist has an important role in this process. In addition, efforts should be made by clinicians to hear and understand the needs and expectations of adolescents before the start of treatment.
Davidson et al^29^ (2012) / Canada / Journal of Canadian Dental Association	Examine the life experiences of women orthodontists about the balance between family and work.		Qualitative and quantitative / Semi-structured interviews / 13 / Adults	The findings reflect their definitions of balance, the specific challenges for the practice of orthodontics, and the strategies developed by women to combine maternal and professional roles to achieve balance.
Davis et al^14^ (2015) / United States of America / American Journal of Orthodontics and Dentofacial Orthopedics	Identify and categorize motivational profiles that explain the reasons why Hispanic or Latino and white parents seek orthodontic treatment for their children, and determine whether there are differences between the parent groups.		Qualitative and quantitative / Interviews / 10 (qualitative) and 70 (quantitative) / Adults	Declarations resulting from the study suggest that four general topics may cover the main reasons for parents seeking orthodontic treatment for their children: sense of responsibility instilled by a professional; need for oral health care perceived by the clinician; preventing future problems or motivation issues for their children; and aesthetic benefit to improve the child’s self-image.
Delalíbera et al^15^ (2010) / Brazil / Acta Scientiarum Health Science	Evaluate the aesthetic results of Class II patients undergoing corrective orthodontic treatment, from the patient’s perspective, and to compare it with normative results obtained by complementary examinations after treatment.		Qualitative and quantitative / Semi-structured interviews / 7 / Adolescents / Adults	The results showed that different facial angles and proportions of what is proposed as scientifically aesthetic does not interfere with the results of treatment, provided that the facial perception of the subjects involved meet the standards of normality and accepted by them and those set by society.
Flett et al^16^ (2014) / United Kingdom / Journal of Orthodontics	Explore the views of potentially orthognathic patients considering the influence of a DVD about orthognathic surgery in the decision to perform the procedure.		Qualitative / Unstructured interviews / 10 / Adolescents / Adults	The DVD provided reliable information that patients do not get or cannot understand from professionals or the internet. If used properly, it can play an important role in the decision to perform the procedure, but should be viewed within a broader context.
Josefsson ^17^ (2010) / Sweden / Swedish Dental Journal (supplement)	Generate a theory that elucidates the complaints of young adults with poor dental aesthetics and the measures they adopt for dealing with this situation on a daily basis.		Qualitative / Unstructured interview / 13 / Adolescents / Adults	A core categorized as “being under the pressure of social norms” was generated and can be applied to improve the understanding of young adults who adjust to poor aesthetics, and to identify those who are not well-adjusted and could benefit from treatment.
Meaney et al^18^ (2012) / Ireland / European Journal of Orthodontics	Determine the impact of congenital absence of teeth on psychosocial and functional well-being and identify important factors for patients that would be incorporated into health status measures for population studies and clinical trials.		Qualitative / Semi-structured interviews / 10 / Adolescents / Adults	Patients have difficulty in understanding their condition and the implications for treatment after the initial diagnosis. The delay between diagnosis and orthodontic and restorative treatment was a common problem.
Pabari et al^19^ (2011) / United Kingdom / American Journal of Orthodontics and Dentofacial Orthopedics	Determine the motivation and expectations of adults for orthodontic treatment and develop a measure to evaluate these motivating factors and psychological traits of these patients.		Qualitative and quantitative / Focus groups / Questionnaire / 25 (qualitative) / 135 (quantitative) / Adolescents / Adults	The desire for aligned teeth was one of the factors reported by the participants, with self-motivation of around 50%. Social pressure was also a factor.
Prabakaran^20^ (2002) / United Kingdom / American Journal of Orthodontics and Dentofacial Orthopedics	Investigate the motivations of adolescents and parents/guardians for orthodontic treatment and try to classify them according to their perceptual similarities.		Qualitative and quantitative / Unstructured interviews / 24 (qualitative) / 120 (quantitative) / Adolescents / Adults	For adolescents, aesthetics was the most significant factor in the search for orthodontic treatment, while for parents/guardians it was concern about the possibility of future problems.
Rachel Henzell et al^39^ (2014) / New Zealand / Angle Orthodontics	Analyze the content of orthodontics-related posts on Twitter		Qualitative / 131 tweets / Adolescents / Adults	Users expressed positive and negative experiences. However, the negatives were offset by the expectation of the aesthetic results achieved.
Ryan et al^21^ (2009) / United Kingdom / Journal of Orthodontics	Develop a measure to assess perceptions of orthognathic patients about referral to a mental health professional.		Qualitative / Semi-structured interviews / 20 / Adults	Development of a questionnaire in which two main issues were addressed: Provision of service and mental health professional’s perceptions.
Ryan et al^22^ (2012) / United Kingdom / American Journal of Orthodontics and Dentofacial Orthopedics	Qualitatively explore and analyze the wide range of impacts of dentofacial deformity, and understand patients’ motivations for seeking orthognathic treatment.		Qualitative / Semi-structured interviews / 18 / Adolescents and Adults	The motivating factors for treatment are directly or indirectly associated with the impact of the condition, which may be related to a complex generated by other factors such as personality, education, and personal relationships.
Shelton et al^23^ (2015) / United Kingdom / Orthodontics & Craniofacial Research	Develop a questionnaire to assess the psychosocial aspects that orthognathic patients considered important regarding their dentofacial deformities.		Qualitative and quantitative / Semi-structured interviews / 30 / Adolescents (over 16 years’ age) / Adults	The specific questionnaire for orthognathic patients proved to be reliable, valid, and sensitive for evaluating the psychological aspects related to dentofacial deformities of these patients, which did not happen with anxiety and depression questionnaires
Soma et al^33^ (2012) / New Zealand / Australian Orthodontic Journal	Investigate the daily practice routine of orthodontists in order to generate an understanding of the reality of the specialty’s practice and its effects on their personal and professional lives.		Qualitative / Semi-structured interviews / 19 / Adults	Demonstrates the value of observing how orthodontists continue to develop in response to changes in society in New Zealand.
Soma et al^35^ (2012) / New Zealand / Australian Orthodontic Journal	Investigate the balance between personal and professional life of orthodontists in New Zealand to generate greater understanding of the effect of professional practice on the personal lives of professionals.		Qualitative / Semi-structured interviews / 19 / Adults	Although New Zealand orthodontists are aware of the need for a balance between personal and professional life, some factors such as the impossibility of reducing the workload and stress related to the profession hinder this process.
Stanford et al^24^ (2014) / United Kingdom / American Journal of Orthodontics and Dentofacial Orthopedics	Examine the concept of dentofacial normality from the perspective of orthodontic patients, using qualitative research methodology.		Qualitative / Semi-structured interviews / 15 / Adolescents / Adults	The constructs of normality consist of personal experiences and can be influenced by external factors such as professionals, friends, media ... Normal appearance seems to include biological and social elements.
Stephens et al^40^ (2013) / United Kingdom / American Journal of Orthodontics and Dentofacial Orthopedics	Investigate how adolescent patients find information about orthodontic treatment, why they seek such information, and what are their preferences for accessing the same.		Qualitative and quantitative / Semi-structured interviews / 15 (qualitative) and 50 (quantitative) / Adolescents	Main means of information: conversation with orthodontist and reading pamphlets. The preferred mode of information was verbal. While most used the internet as a social network, it was not used as expected.
Taghavi Bayat et al^25^ (2013) / Sweden / Acta Odontológica Scandinavica	Explore how malocclusions affect the daily lives of adolescents and how they deal with the afflictions related to them.		Qualitative / Focus groups / 12 / Adolescents	Patients appeared repeatedly reminded of their dental conditions. Low self-esteem was reinforced by media influences.
Twigge et al^27^ (2016) / Australia / European Journal of Orthodontics	Evaluate, in the short- and long-term, orthodontic treatment expectations, malocclusion severity, and oral health-related quality of life status of adolescent patients		Qualitative and quantitative / Interviews / 105 / Adolescents (12-17 years of age)	Female patients tend to experience worse psychosocial impacts related to their malocclusions, compared to males with the same need for orthodontic treatment. Adolescent patients seek to improve their dental appearance and aspects of their quality of life.
Twigge et al^41^ (2016) / Australia / American Journal of Orthodontics and Dentofacial Orthopedics	Assess, through patient facial images and qualitative methodology, patients’ orthodontic concerns, which are incorporated into and are important in treatment planning and consent.		Qualitative and quantitative / Interviews / 105 / Adolescents (12-17 years old)	Adolescents were more concerned with the appearance of crowding and gaps in the front teeth, and with the difficulty of cleaning these teeth. The display of facial images helped the teenagers to identify additional concerns related to appearance.
Veeroo et al^37^ (2014) / United Kingdom / American Journal of Orthodontics and Dentofacial Orthopedics	Conduct a pilot test to evaluate the effectiveness of ways of evaluating complaints about the use of intermaxillary elastics and investigate the motivation for their use.		Qualitative and quantitative / Semi-structured interviews / 14 / Adolescents	The questionnaire evaluated showed no difference in the normal routine instructions on the use of elastics, although it showed a tendency for better use.
O’Keeffe et al^30^ (2016)/ United Kingdom / Journal of Orthodontics	Evaluate the experiences of patients with hypodontia in a teaching hospital, after diagnosis, and then their satisfaction with the orthodontic care received and the result at the end of active orthodontic treatment.		Qualitative / Unstructured and semi-structured interviews / 20 / Adolescents and adults	The importance of patient reporting was highlighted in all interviews. The main areas that could be strengthened were related to the importance of ensuring good communication, particularly with individuals undergoing complex multidisciplinary treatment.
Gassem et al^31^ (2016) / United Kingdom / Journal of Dentistry	Design and evaluate the psychometric properties of a measure investigating the expectations of patients with hypodontia, concerning the process and outcome of combined orthodontic/restorative treatment.		Qualitative and quantitative / Interviews / 41 (qualitative) and 42 (quantitative) / Adolescents and adults	A patient-based measure for the process and outcome of combined orthodontic-restorative treatment was developed for patients with hypodontia. It had good validity and satisfactory internal consistency.
Kearney et al^32^ (2016)/ United Kingdom / American Journal of Orthodontics and Dentofacial Orthopedics	Identify occlusal discrepancies related to the anterior mandibular segment that may lead to a decision to undergo orthodontic retraction.		Qualitative and quantitative / Focus groups / Questionnaire / 50 50 / Adolescents and adults	The perception of mandibular anterior segment irregularity and its influence on the need for orthodontic retraction are complex and multifaceted. However, the horizontal discrepancies of the incisors were considered the most significant by both lay and professional evaluators.
Jones et al^34^ (2016) / United Kingdom / European Journal of Dental Education	Explore undergraduate student experiences in the treatment of orthodontic emergencies within an educational institution.		Qualitative / Semi-structured interviews / 72 / Adults	Most students were confident in taking charge of orthodontic emergencies. The theoretical knowledge supplemented by exposure to a range of clinical problems within a learning environment made the students feel more confident.

### Description of the studies

**Figure 2:**
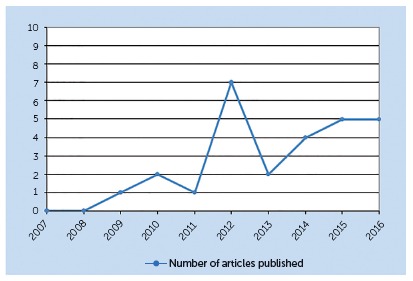
Growth in the number of qualitative research publications on orthodontics in the 10-year period (2007-2016).

The publications showed a trend to increase over the last four years (2013-2016), concentrating almost 60% of the publications (16 articles) (Fig 2).

A descriptive summary of the main characteristics of the reviewed articles is presented in Table 2.

**Table 2:** Summary description of the main characteristics of the reviewed articles.

Source	Journal	Object of study	Methodology used	Subjects of the studies	Data collection method
» United Kingdom (n=15) » Sweden (n=3) » New Zealand (n=3) » Australia (n=2) » Canada (n=1) » Ireland (n=1) » USA (n=1) » Brazil (n=1)	AJO-DO (n=10) J. Orthod (n=3) Eur J Orthod (n=3) Orthod Craniofac R (n=2) Austr Orthod J (n=2) Acta Od Scand (n=1) JCDA-CA (n=1) Angle Orthod (n=1) Swed J D (n=1) Acta Scient Health (n=1) J Dent (n=1) Eur J Dent Educ (n=1)	» Perceptions related to malocclusion and orthodontic treatment (n=17) » Clinical practice of orthodontics (n=4) » Behavior in orthodontic treatment (n=3) » Assessment of information about orthodontic treatment (n=3)	» Qualitative (n=15) » Qualitative and quantitative (n=12)	» Adolescents (n=7) » Adults (n=6) » Adolescents + Adults (n=14)	» Semi-structured interview (n=13) » Non-structured interview (n=8) » Semi-structured + Non structured (n=1) » Focus group (n=1) » Focus group + questionnaire (n=2) » Posted net content (n=2)

The qualitative studies were mostly carried out in Europe, with 19 publications, originating from the United Kingdom (15 articles), followed by Sweden (3 articles), and Ireland (1 article).

### Objects of the studies

Seventeen reviewed articles (63%) had as their object the perception of people regarding their motivations for orthodontic treatment, about aesthetic and psychosocial impact of malocclusion or orthodontic treatment, and the implications of these factors for their quality of life.^13^^-^^32^

Four studies evaluated aspects related to the clinical practice of orthodontics,^28^^,^^29^^,^^33^^,^^34^ three articles assessed attitudes and behaviors related to orthodontic treatment,^35-37^ and three articles assessed the form and content of information about orthodontic treatment.^38^^-^^40^

### Participants profile

A total of 7 studies were conducted exclusively with adolescent participants, 14 with adults and adolescents, and 6 with adults.

### Designs of the studies

Considering the design of the studies, from the 27 articles analyzed, 15 (55%) used qualitative research methodology exclusively, and 12 (45%) had an association with quantitative methods.

The most commonly used data collection method was the semi-structured interview (13 articles).^15^^,^^18^^,^^21^^-^^24^^,^^28^^,^^29^^,^^33^^-^^35^^,^^37^^,^^40^ Unstructured or open interviews were conducted in 8 studies.^14,16,17,20,27,31,36,41^ and combined with a semi-structured interview in only one study.^30^ The focus group was used exclusively in only one study^25^ and combined with questionnaires in two studies.^19^^,^^32^ Two studies conducted content analyses on information posted on the internet and related to orthodontics.^38^^,^^39^

## DISCUSSION

The quantitative and qualitative research methods are different in nature, from their conception to their final wording for publication, no contradiction nor continuity between them is observed. The first aims to elucidate data, indicators, and tendencies, using large collections of data in the form of variables that will be classified and interpreted.^42^ The second is normally used when little is known about the object of the study and allows the researcher to explore, more intensely, meanings and interpretations of facts, and particular and specific processes rarely observed in quantitative research.^43^ The term “qualitative assessment” is also used in several clinical and laboratory studies to evaluate the performance of devices or techniques without, however, having any relation to the research method.

Due to their particular characteristics, both methodologies in isolation may, in certain circumstances, be insufficient for contemplating all that is actually observed. Extremely detailed descriptions of all the facts known based on human subjectivity may not render a useful mathematical representation. On the other hand, the use of sophisticated mathematical resources for numerical calculations of all coefficients can be completely fruitless if many facts relevant to the problem remain unknown.^44^ Therefore, they can and should be used as complementary approaches, as observed in a number of studies of this review.^14^^,^^15^^,^^19^^,^^20^^,^^23^^,^^27^^,^^28^^,^^31^^,^^32^^,^^37^^,^^40^^,^^39^

Although in recent years there has been an increased demand for the facilitation and dissemination of qualitative research projects in health,^10^ they have until now been underutilized in dentistry.^45^ This is due mainly to the existing historical link of biomedical and clinical research with epidemiology and quantitative methodology, based mainly on the great potential of statistical procedures, capable of generating representative data of a given population.^1^^,^^5^ However, the subjectivity of the factors related to malocclusion and to orthodontic treatment, as well as their impact on quality of life, has been growing in proportion to the importance placed on aesthetics in social, affective, and behavioral relationships in contemporary society. Yet studies in orthodontics have been more relevant for professionals without necessarily considering the values of the patients.^9^ Clinical judgment is based not only on experimental evidence, but also on a subjective assessment by the clinician which is formulated through interpretative interaction, communication, empathy, and experience.^46^ Thus, even if slowly, orthodontics shows signs of understanding the importance of other methodological possibilities for research. Qualitative research offers the potential to explore social and cultural attitudes toward orthodontic treatment, and helps in understanding how people interpret the importance of dental appearance in their lives.^47^ This was confirmed in this review, where 17 articles had their objectives based on this indication of method.

The diversity of study objects presented in this review demonstrates the great potential of qualitative research methods to enrich the knowledge of this specialty, addressing aspects that are impossible to evaluate through quantitative investigation.^43^^,^^47^

One of the main characteristics of the qualitative approach is the study, in amplitude and in depth, of specific phenomena, facts, and processes of delimited groups that can be covered comprehensively.^5^ This may explain the absence of studies with children, who may present greater difficulties in fitting the particularities of the methodology.

The same diversity found in the objects of study also occurred in relation to the data collection methods. The interview was the method of choice for the majority (80%) of the studies evaluated. An interview can be, on one hand, completely structured, or on the other, totally open in the form of a free conversation. However, the collection method most commonly used by qualitative researchers in general is the semi-structured interview. These interviews involve a series of open questions based on pre-established thematic areas covering various topics according to the researcher’s interest. Although the questions under investigation are pre-defined, they allow both the interviewer and interviewee to deepen the discussion on topics of greater interest.^48^

Another collection method often used in qualitative research is the focus group, which, as observed in this study, can be used exclusively^25^ or combined with individual interviews^49^ and questionnaires.^19^ The focus group is a data collection strategy that is characterized as a group interview, usually with six to ten participants, lasting about an hour and a half.^50^ It is based essentially on the interaction among the participants at the time they respond to the topics raised by the researcher, thus influencing the ideas expressed to one another, which would not be possible in an individual interview.^48^^,^^51^

Unlike quantitative research, qualitative investigation does not previously define a total number of participants in the study. This is determined strategically depending on the subject matter and the approach to be adopted, with the recurrence of information as a determining factor of the theoretical saturation of the study topic.^48^

The current study presents important limitations that must be considered. The objective was to demonstrate the applicability of qualitative research in Orthodontics. The diversity of the studied objects, as well as the methods used, makes interpretive validation difficult via comparison of their results. However, the present review clearly shows the increase in the number of qualitative studies and the possible contributions of these methods to the growth of the specialty.

## CONCLUSION

» The orthodontic publications using qualitative methodology have systematically increased in recent years.
» The studies in orthodontics using qualitative methods originate mainly on the European continent and particularly in the UK.
» The studies have as a target participants, preferably adolescents and adults, addressing subjective aspects related to malocclusion and to orthodontic treatment.
» Because of its potential to explore behaviors and socio-cultural attitudes sustained in subjectivity, which are increasingly relevant to the specialty, qualitative research offers new possibilities for orthodontic studies, and may be used in an exclusive or complementary way in relation to quantitative methods.
